# Blood transfusion practices in Africa: advances, barriers, and ways of overcoming the barriers – a narrative review

**DOI:** 10.1097/MS9.0000000000003898

**Published:** 2025-09-15

**Authors:** Emmanuel Ifeanyi Obeagu

**Affiliations:** Department of Biomedical and Laboratory Science, Africa University, Zimbabwe

**Keywords:** Africa, blood safety, blood transfusion, donor recruitment, health care infrastructure

## Abstract

Blood transfusion is a crucial component of health care in Africa, providing life-saving support for patients with severe anemia, trauma, and maternal hemorrhage. Over the years, significant advancements have been made, including improved donor recruitment strategies, enhanced screening technologies, and better infrastructure for blood banking. Innovations such as mobile donor tracking applications and drone technology for blood delivery have further improved accessibility, particularly in remote areas. Policy reforms and international collaborations have also played a key role in strengthening national blood transfusion services across the continent. Despite these advancements, Africa continues to face major challenges in ensuring a safe and sufficient blood supply. Persistent issues such as low voluntary blood donation rates, high prevalence of transfusion-transmissible infections, and inadequate health care infrastructure hinder progress. Financial constraints, workforce shortages, and weak regulatory frameworks further compromise the efficiency and sustainability of blood transfusion services. These barriers contribute to a continued reliance on family replacement donors and, in some cases, unsafe transfusion practices that pose risks to patients.

## Introduction

Blood transfusion is a critical medical intervention that saves millions of lives globally by managing severe anemia, hemorrhage, trauma, and various blood disorders^[1,2]^. In Africa, where conditions such as sickle cell disease, malaria-induced anemia, and postpartum hemorrhage are prevalent, the demand for safe and timely blood transfusion services is exceptionally high^[3,4]^. However, ensuring an adequate and sustainable blood supply remains a persistent challenge, influenced by factors such as socio-cultural perceptions, health care infrastructure, and economic constraints. Despite these challenges, significant strides have been made in improving blood transfusion services in several African countries[5]. Advances in blood donor recruitment, implementation of modern screening techniques, and the integration of innovative technologies have enhanced blood safety and accessibility. Many nations have shifted from relying on family replacement donors to voluntary non-remunerated blood donation (VNRBD), which is associated with a lower risk of transfusion-transmissible infections (TTIs). Moreover, mobile applications for donor tracking and drone-based blood delivery have emerged as innovative solutions to logistical barriers in remote areas^[6,7]^. International collaborations and policy reforms have also contributed to progress in Africa’s blood transfusion landscape. Organizations such as the World Health Organization (WHO), the African Society for Blood Transfusion (AfSBT), and the Global Fund have provided technical and financial support to strengthen national blood transfusion services. These efforts have led to the establishment of centralized blood banks, standardized screening protocols, and improved regulatory frameworks. However, the impact of these initiatives varies across different regions due to disparities in health care infrastructure and government commitment^[8,9]^.

**HIGHLIGHTS**

- **Advances in Safety:** Improved screening methods enhance transfusion safety and reduce infectious risks.
- **Barriers to Access:** Limited blood donation culture and infrastructure hinder availability.
- **Logistical Challenges:** Inadequate storage and distribution systems affect timely access.
- **Innovative Solutions:** Mobile blood banks and donor education boost supply.
- **Policy Improvements:** Strengthening regulations and funding enhances sustainability.

Despite these advancements, the blood supply in Africa remains insufficient to meet the growing demand. Many countries experience chronic blood shortages due to low donor participation, largely driven by cultural beliefs, religious concerns, and misconceptions about blood donation. Additionally, seasonal fluctuations in blood availability, particularly during malaria outbreaks, further strain blood bank reserves. Without sustained efforts to promote voluntary blood donation, many patients continue to rely on emergency family replacement donors or, in critical cases, go without necessary transfusions^[10–12].^ Another major challenge in African blood transfusion services is the high prevalence of TTIs, including HIV, hepatitis B and C, and syphilis. Although many countries have introduced advanced screening techniques, such as nucleic acid testing (NAT) and enzyme-linked immunosorbent assays (ELISA), these methods are not widely available due to financial and infrastructural constraints. In some rural areas, outdated screening procedures or lack of proper quality control measures increase the risk of unsafe transfusions, further exacerbating public health concerns^[13–16].^ Financial limitations also pose a significant barrier to the sustainability of blood transfusion services in Africa. Blood collection, testing, storage, and distribution require substantial investment, yet many health care systems operate with limited funding. Many African countries rely on external donors for financial support, making long-term sustainability uncertain. Additionally, the shortage of trained health care professionals, including transfusion specialists and laboratory technicians, hinders the efficient operation of blood banks and transfusion services[17].

## Aim

This review aims to examine the current state of blood transfusion practices in Africa, highlighting the significant advancements made in donor recruitment, blood screening technologies, and infrastructure development.

## Review methods

The methodology for this review on “Blood Transfusion Practices in Africa: Advances and Barriers” was developed to ensure a comprehensive and systematic approach to gathering, analyzing, and synthesizing relevant literature. The following outlines the steps taken to conduct this review.

### Literature search strategy

A thorough literature search was conducted to gather information from peer-reviewed journals, books, government reports, and reputable health care organization publications. Key databases, including PubMed, Google Scholar, ScienceDirect, and JSTOR, were searched for articles published between 2000 and 2024, focusing on blood transfusion practices, challenges, and advancements in Africa. The search terms used included “blood transfusion in Africa,” “blood donation challenges Africa,” “transfusion-transmissible infections Africa,” “blood transfusion infrastructure Africa,” and “blood safety practices Africa.”

### Inclusion and exclusion criteria

To ensure the relevance and quality of the selected studies, the following inclusion criteria were applied:

- Studies and reports focusing on blood transfusion practices in African countries.
- Research articles discussing both advances and challenges in the field.
- Literature published in English.
- Studies published within the past two decades to ensure the inclusion of the most current information.

**Exclusion criteria included:**

- Studies focusing solely on non-African countries.
- Research that did not address either the advancements or barriers related to blood transfusion practices.
- Articles that lacked data on practical solutions to overcoming the identified barriers.

## Recent advances in blood transfusion practices in Africa

In recent years, several African nations have adopted transformative approaches in blood transfusion practices, marked by technological integration, system centralization, and digital innovations. These developments have contributed significantly to improving patient outcomes, particularly in reducing TTIs and enhancing the response to obstetric and surgical emergencies[18].

### Introduction of nucleic acid testing (NAT)

One of the most critical advancements in transfusion safety has been the rollout of NAT in select countries such as South Africa, Botswana, and Kenya. NAT allows for earlier detection of viral pathogens (e.g., HIV, HBV, HCV) during the window period, significantly reducing the risk of TTIs compared to conventional serological tests[19].

- In South Africa, the South African National Blood Service (SANBS) implemented NAT as a standard screening tool. As a result, the residual risk of HIV transmission through transfusion has dropped to less than 1 in 1.5 million donations – among the lowest globally.
- Botswana, leveraging support from PEPFAR, has adopted NAT alongside serology in high-prevalence areas, contributing to a measurable reduction in TTI rates among transfusion recipients[20].

### Centralization of blood systems

The transition from fragmented, hospital-based blood collection to centralized, coordinated national blood systems has led to more consistent standards in donor screening, blood processing, and quality control.

- Rwanda’s National Centre for Blood Transfusion (NCBT) exemplifies this shift, operating a hub-and-spoke model with centralized testing and storage facilities linked to regional hospitals. This model has improved inventory management and reduced delays in emergency transfusions, particularly for women experiencing postpartum hemorrhage[21].
- Namibia, through its Blood Transfusion Service of Namibia, has successfully implemented a national system relying solely on voluntary nonremunerated blood donors. This has resulted in improved donor safety profiles and fewer reported cases of TTIs in recipients[22].

### Implementation of digital traceability systems

Digital innovations have enhanced traceability, accountability, and data-driven decision-making in blood services:

- South Africa utilizes a digital donor management platform that tracks donation histories, test results, and inventory flow. This has improved donor retention, ensured rapid removal of infected units, and optimized the matching of blood types to patient needs.
- Botswana has integrated transfusion data into its national health information system, enabling real-time tracking of blood usage and facilitating audits of transfusion-related adverse events[23].

These systems also allow for better forecasting of demand, reducing waste due to expired blood and ensuring equitable distribution to underserved areas.

### Impact on patient outcomes

The adoption of these advances has yielded tangible clinical benefits:

- **Reductions in TTIs:** Countries that have implemented NAT and centralized screening – such as South Africa and Namibia – report significantly lower rates of transfusion-related HIV, HBV, and HCV infections compared to regional averages.
- **Improved Maternal Outcomes:** Rwanda’s centralized and drone-supported blood delivery system has been credited with reducing maternal mortality from obstetric hemorrhage, especially in remote rural districts.
- **Faster Emergency Response:** The digitization of inventory systems in Botswana and South Africa enables rapid mobilization of rare blood types during trauma or surgery, improving survival rates in critical care cases[24].

## Barriers to effective blood transfusion practices in Africa

### Low voluntary blood donation rates

One of the most significant challenges in Africa’s blood transfusion services is the low rate of voluntary, non-remunerated blood donation (VNRBD). Many African countries still rely heavily on family replacement donors, which are not a sustainable model and poses safety risks. Cultural beliefs, religious concerns, fear of needles, and misconceptions about blood donation contribute to reluctance among potential donors. Additionally, a lack of consistent public awareness campaigns and donor education further hinders efforts to establish a stable and reliable donor pool^[25–27]^.

### High prevalence of transfusion-transmissible infections (TTIs)

The risk of TTIs remains a major challenge in Africa due to the high prevalence of diseases such as HIV, hepatitis B and C, and syphilis. Despite improvements in screening technologies, not all health care facilities have access to advanced diagnostic tools like NAT and ELISA. In some rural and underfunded areas, outdated screening methods or inadequate quality control measures increase the risk of unsafe transfusions. Ensuring universal access to high-quality screening remains a significant hurdle[28].

### Inadequate blood bank infrastructure

Limited infrastructure for blood collection, storage, and distribution is a major barrier to effective blood transfusion services in Africa. Many regions face challenges such as insufficient cold chain storage systems, lack of blood transportation facilities, and poor coordination between blood banks and health care institutions. In rural and remote areas, the absence of centralized blood banking systems often results in delays in accessing safe blood, leading to preventable deaths. Additionally, frequent power outages in some countries further compromise blood storage and preservation[15].

### Financial constraints and sustainability issues

Blood transfusion services require substantial financial investment, but many African health care systems operate under severe budgetary constraints. Costs associated with blood collection, testing, storage, and distribution are often beyond the reach of public health budgets, leading to reliance on external donors and international organizations for funding. This dependence on foreign aid makes long-term sustainability uncertain. Additionally, many patients struggle to afford the costs of transfusion services, particularly in private health care settings where blood is often sold at high prices[15].

### Shortage of skilled health care personnel

A lack of trained health care professionals, including transfusion specialists, laboratory technicians, and blood bank personnel, affects the efficiency of blood transfusion services in Africa. Many health care workers receive limited training in transfusion medicine, leading to improper handling, storage, and administration of blood products. Furthermore, the migration of skilled professionals to higher-income countries exacerbates the shortage, leaving many hospitals understaffed and unable to provide timely and safe transfusion services. Expanding training programs and retaining skilled personnel are essential to improving blood transfusion practices[16].

### Weak regulatory frameworks and policy implementation

While many African countries have developed national blood policies, the implementation and enforcement of these regulations remain weak. Inconsistent policies on blood donor recruitment, screening standards, and transfusion protocols lead to variations in blood safety across different regions. Corruption and mismanagement in some health care systems also undermine efforts to improve blood transfusion services. Strengthening regulatory frameworks, ensuring compliance with international standards, and improving governance in blood transfusion management are necessary to address these challenges[28].

### Logistical challenges and geographic barriers

The vast geographic landscape of Africa presents logistical difficulties in ensuring timely and equitable blood distribution. Many rural areas lack accessible roads, making it challenging to transport blood to health care facilities in emergencies. Although some countries have adopted drone technology for blood delivery, this innovation is not yet widespread. Seasonal factors, such as flooding and drought, can further disrupt supply chains, leading to critical shortages in high-demand regions. Addressing these logistical barriers requires investment in transportation infrastructure and alternative distribution strategies[27].

## Region-specific barriers in blood transfusion systems across Africa

Despite shared continental challenges, blood transfusion services across Africa are not uniform. Significant regional variations exist, shaped by economic capacity, political commitment, health care infrastructure, and sociocultural dynamics. Comparing well-resourced systems such as those in South Africa and Rwanda with more fragmented or underfunded systems in parts of Central and West Africa reveals critical disparities that directly influence the safety, availability, and accessibility of blood products[25].

### Infrastructure and technological disparities

In countries like South Africa, robust infrastructure enables sophisticated practices such as universal NAT, comprehensive cold chain systems, and automated blood tracking platforms. Rwanda has also invested heavily in centralized facilities and innovative delivery mechanisms (e.g., drone delivery to rural areas), ensuring timely access to safe blood products[26]. In contrast, many West African and Central African nations (e.g., Guinea, Chad, Central African Republic) operate under resource constraints that limit blood safety and distribution:

- **Lack of Cold Chain and Transport:** In rural areas of Niger and Burkina Faso, blood units are often stored in nonrefrigerated conditions or transported over long distances without temperature control, compromising safety.
- **Limited Testing Capacity:** Some countries lack reliable laboratory infrastructure for TTIs screening, relying on rapid diagnostic tests with lower sensitivity, especially in peripheral health facilities.

### Policy and governance gaps

Policy implementation is another critical area where regional differences are stark:

- South Africa and Rwanda have national blood policies, dedicated regulatory bodies, and standardized protocols enforced across public and private sectors. These countries have also succeeded in transitioning to VNRBD models, in line with WHO recommendations.
- Conversely, countries like Sierra Leone, Liberia, and Democratic Republic of Congo (DRC) struggle with weak policy frameworks, limited regulatory oversight, and dependence on family replacement or paid donors, which are associated with higher rates of TTIs and donation delays[27].

In many underfunded systems, blood services are fragmented across regional hospitals without central coordination, resulting in supply mismatches and inconsistent screening standards.

### Rural-urban disparities in access and quality

Urban centers often have better-equipped hospitals and centralized blood banks. For instance:

- In Lagos (Nigeria) or Nairobi (Kenya), tertiary hospitals may have on-site blood banks with screening and storage capacity.
- However, in rural areas, particularly in northern Nigeria, eastern Cameroon, and rural Ethiopia, patients may be asked to provide family donors or wait for blood to be transported from distant centers[28].

This urban bias exacerbates maternal mortality and trauma-related deaths in remote areas where emergencies demand immediate transfusion support.

Additionally, cultural beliefs and misconceptions about blood donation are more pronounced in rural communities, where outreach and education campaigns are often lacking or inconsistently applied.

### Workforce and training challenges

Well-resourced systems benefit from trained transfusion specialists, lab technologists, and quality assurance teams. In under-resourced regions:

- Human resource shortages persist, with transfusion services often staffed by general practitioners or nurses without specific training.
- Limited opportunities for continuing medical education contribute to inconsistent adherence to best practices and guidelines[29].

For example, Cameroon and Guinea-Bissau report gaps in personnel training, resulting in improper cross-matching, poor hemovigilance, and inadequate adverse event reporting.

## Practical ways of overcoming barriers to blood transfusion in Africa

### Increasing voluntary blood donation

Governments and health organizations should conduct regular nationwide blood donation awareness campaigns using social media, radio, television, and community outreach programs to dispel myths and encourage donation. Offering nonmonetary incentives such as certificates, donor appreciation events, and free health checkups can encourage voluntary blood donation. Schools, universities, religious institutions, and workplaces can be engaged to organize regular blood donation drives and integrate blood donation education into their programs. Deploying mobile blood collection centers in high-traffic areas such as markets, workplaces, and schools can increase donor participation and convenience^[29,30]^.

### Strengthening screening and blood safety measures

Governments should prioritize the acquisition of NAT and ELISA machines to enhance the detection of TTIs. Implementing strict quality control measures and continuous monitoring of screening processes will help maintain high blood safety standards. Continuous professional training for laboratory personnel on proper blood handling, screening, and storage techniques is crucial to minimizing errors and ensuring safe transfusions^[10,31]^.

### Expanding blood bank infrastructure and distribution systems

Investment in modern cold chain storage, improved blood processing equipment, and reliable electricity supply (including solar-powered refrigeration systems) will help maintain a steady blood supply. Creating well-equipped regional blood banks will decentralize blood collection and distribution, ensuring that even remote areas have access to safe blood. Countries like Rwanda and Ghana have successfully used drones to deliver blood to remote areas. Expanding this technology across Africa can significantly reduce delays in blood supply[32].

### Securing sustainable funding and financial support

Governments should prioritize blood transfusion services in their national health care budgets to ensure sustainable funding. Collaborations between governments, non-governmental organizations (NGOs), and private sector stakeholders can provide additional financial and technical support. Implementing cost-effective screening methods and efficient blood management systems can help reduce wastage and optimize available resources[23].

### Increasing skilled workforce and training programs

Establishing specialized training programs in transfusion medicine for health care workers will help improve the efficiency of blood transfusion services. Offering competitive salaries, career development opportunities, and incentives for health care workers can help prevent brain drain and retain skilled professionals. Including transfusion medicine in medical and nursing school curricula will ensure that future health care professionals are well-equipped with the necessary skills[33].

### Strengthening regulatory frameworks and policy implementation

Standardizing blood transfusion guidelines across African countries will help ensure uniform safety measures and best practices. Governments should establish independent regulatory bodies to oversee blood transfusion services and enforce quality control measures. Partnering with organizations like the WHO and the AfSBT can provide technical expertise and resources to improve blood transfusion services[34].

### Addressing geographic and logistical barriers

Developing better road networks and emergency transport services will improve the timely delivery of blood, especially in rural areas. Deploying mobile blood collection and distribution units to underserved regions can help bridge the gap in blood supply. The use of mobile applications for blood donor tracking, emergency alerts, and inventory management can improve efficiency in blood supply and distribution[32].

## Regional innovations, transfusion safety, and policy reforms in Africa

Blood transfusion systems in Africa are undergoing progressive changes driven by regional innovations, heightened attention to transfusion safety, and strategic policy reforms. Although disparities persist across countries, notable improvements have emerged in several regions, demonstrating that context-specific interventions can substantially enhance the safety, accessibility, and efficiency of blood services.

### Regional innovations and technological integration

Several African nations have embraced innovative technologies and operational models to improve blood collection, processing, and distribution:

- **Rwanda** has garnered global recognition for its partnership with Zipline, a drone logistics company that delivers blood to remote health facilities within minutes. This innovation has significantly reduced maternal mortality due to postpartum hemorrhage by ensuring timely access to blood products, particularly in rural areas[1].
- **South Africa**, through the SANBS, has implemented advanced data systems for donor tracking, blood inventory management, and real-time surveillance of blood usage trends. SANBS also employs NAT for early detection of TTIs, setting a regional benchmark for safety.
- **Namibia** has achieved full VNRBD through targeted educational campaigns and integration of donor mobilization within schools and workplaces. Namibia also utilizes centralized screening facilities to ensure high-quality testing and reduce TTIs[30].

### Enhancing transfusion safety and quality control

Despite high TTI prevalence in parts of sub-Saharan Africa, some countries have made significant strides in blood safety:

- **Kenya** introduced rigorous screening protocols with the support of the U.S. President’s Emergency Plan for AIDS Relief (PEPFAR), focusing on HIV, HBV, HCV, and syphilis testing using ELISA and NAT.
- In **Ghana**, quality assurance programs are now embedded in regional blood centers to standardize practices and improve traceability, supported by capacity-building efforts from the WHO and regional partners.
- **Botswana** integrates blood safety monitoring with its broader HIV/AIDS control program. This alignment has enabled a consistent supply of screened blood and minimized the risk of TTIs, particularly among high-transfusion populations such as children with sickle cell disease and women with obstetric complications[31].

### Policy reforms and strategic national planning

Policy-level reforms are increasingly recognized as critical levers for sustainable improvement in transfusion services:

- **Nigeria** revised its National Blood Policy in recent years to prioritize centralized coordination, with plans to establish a National Blood Service Commission. The reform aims to harmonize blood banking standards across its federated system, curb commercial blood donations, and promote voluntary blood drives[4].
- **Ethiopia** has implemented a five-year National Blood Transfusion Strategy focusing on infrastructure strengthening, decentralization of services, and public awareness campaigns. As a result, voluntary donation rates have steadily increased, and the dependence on family replacement donors has declined.
- **Uganda**, in collaboration with the WHO, has developed a National Strategic Plan for Blood Safety that includes community engagement, integration with emergency obstetric care, and investments in cold chain logistics for rural areas[10].

### Regional collaboration and institutional support

Regional bodies such as the AfSBT play a vital role in standardizing practices across the continent. Through accreditation programs and technical training, AfSBT supports countries in achieving compliance with international blood safety standards. Moreover, partnerships with international donors (e.g., PEPFAR, Global Fund, GAVI) have provided essential funding for equipment, testing kits, and staff training. However, sustainability remains a concern, underscoring the need for increased domestic investment and policy continuity[32].

## Strategies to improve blood donor recruitment and testing efficiency in Africa

Strengthening the blood supply chain in Africa requires more than technical advances; it demands innovative and context-sensitive strategies for recruiting donors and enhancing testing processes. In the revised manuscript, we analyze the relative effectiveness of several approaches – notably community-based blood drives, mobile blood collection units, and public education campaigns – using real-world data from Kenya, Ghana, and Uganda (Table 1)[33].

**Table 1:** Country-level blood transfusion strategies and reported outcomes

Country	Key Strategies	Outcomes/Impact
Rwanda	Centralized blood donation system; NAT rollout	Increased voluntary donations by 30%; Reduced TTIs by 40%
South Africa	Digital traceability; extensive donor education	Improved blood safety; > 90% voluntary donations; TTI rates <1%
Namibia	Mobile collection units; health worker training	Expanded rural donor base by 25%; Enhanced staff competencies
Uganda	Mobile units; community sensitization campaigns	20% increase in donor numbers; Improved testing efficiency
Ghana	Public-private partnerships; subsidized transport	Strengthened supply chains; Reduced donor dropout rates
Kenya	Integration with HIV programs; data systems	Improved donor screening; Better inventory management

### Community-based blood drives

Community-led donor mobilization has shown significant promise in expanding the donor base, especially in areas where institutional blood donation is limited.

- In Kenya, partnerships between the Kenya National Blood Transfusion Service (KNBTS) and religious institutions, local chiefs, and schools have increased donation rates in rural and peri-urban areas. A 2022 national report noted a 17% increase in voluntary blood donors in counties with consistent community outreach programs compared to regions without them.
- Similarly, Ghana’s National Blood Service works closely with community health workers and civic organizations to organize weekend and market-day blood drives, resulting in higher engagement from first-time donors[34].

These strategies are most effective when paired with culturally sensitive messaging that addresses misconceptions about blood donation and emphasizes its lifesaving potential.

### Mobile blood collection units

Deploying mobile units to reach donors in underserved or geographically distant areas helps mitigate access barriers and supports rapid replenishment of blood stocks.

- Uganda’s Blood Transfusion Service has expanded its mobile unit fleet to serve both urban slums and remote regions. In 2021 alone, mobile collections accounted for over 60% of the national blood supply, with regions like Mbale and Gulu reporting 30% fewer stockouts in emergency situations compared to previous years.
- In Ghana, mobile units equipped with solar-powered refrigeration and point-of-care testing technologies have improved collection logistics and preserved sample quality during transport, especially in northern regions[32].

Mobile drives are particularly impactful when synchronized with national campaigns (e.g., World Blood Donor Day), leveraging momentum and media visibility.

### Public education and awareness campaigns

Educational campaigns remain a cornerstone of sustained donor engagement, particularly among youth and low-income populations.

- In Kenya, school-based blood donation awareness initiatives combined with mobile outreach in universities led to a 25% rise in youth donors (ages 18–25) over a 3-year period[3].
- Uganda launched a mass media campaign in 2022 featuring testimonials from transfusion beneficiaries, resulting in a measurable spike in voluntary donations during the first quarter of the year.
- Ghana’s Ministry of Health has integrated blood donation messages into routine antenatal care education and community radio programs, helping normalize voluntary donation across age and gender groups[32].

These campaigns are more successful when messages are locally tailored, multilingual, and accompanied by practical opportunities to donate.

### Improved testing efficiency

Strategic reforms in testing workflows and equipment use have also contributed to better blood safety outcomes:

- Uganda introduced centralized ELISA-based testing hubs, reducing test turnaround time by 40% and enabling faster clearance of collected blood units for clinical use.
- In Kenya, the integration of digital donor registries has streamlined the matching of test results with donors, aiding in prompt deferral of high-risk individuals and improving traceability.

Outcome data from both countries show increased testing throughput, reduced discard rates, and more timely availability of safe blood, which has directly impacted care quality in maternal health and trauma settings.

## Strategic partnerships in strengthening blood transfusion services in Africa

The development and sustainability of effective blood transfusion systems across Africa are strongly supported by multi-stakeholder partnerships. Collaboration between governmental health ministries, international organizations like the WHO, PEPFAR, and various non-governmental organizations (NGOs) has been instrumental in addressing the systemic gaps in blood services – from infrastructure and workforce development to policy guidance and donor mobilization.

### WHO and global policy guidance

The WHO plays a central role in providing strategic direction and technical frameworks to African countries striving to align with global standards for blood safety.

- WHO’s “Action Framework to Advance Universal Access to Safe, Effective and Quality-Assured Blood Products 2020–2023” has guided many African nations in developing national blood policies, hemovigilance systems, and quality assurance frameworks.
- Through its regional offices, WHO also conducts training programs, supports capacity-building for national blood transfusion services, and provides data collection tools to monitor blood donation, testing, and utilization[35].

### PEPFAR and HIV-safe blood initiatives

The U.S. President’s Emergency Plan for AIDS Relief (PEPFAR) has made significant investments to reduce the risk of HIV transmission through blood transfusions in Africa.

- PEPFAR has supported the strengthening of transfusion laboratories, training of blood safety personnel, and expansion of VNRD recruitment programs.
- In countries like Mozambique, Tanzania, and Lesotho, PEPFAR helped establish centralized testing facilities and introduced NAT to enhance detection of TTIs[36].

These efforts have not only improved blood safety but have also contributed to broader public health system strengthening.

### National governments and health ministries

African governments are increasingly recognizing the importance of safe and adequate blood supplies as part of universal health coverage and emergency preparedness strategies.

- Rwanda and Namibia have adopted centralized blood services under the direct oversight of their ministries of health, ensuring uniform protocols, coordinated donor recruitment, and streamlined resource allocation.
- Countries like Uganda and Ethiopia have established national blood transfusion councils, often working in conjunction with NGOs and academic institutions to train health care workers and expand donor outreach[37].

### NGOs and civil society engagement

Non-governmental organizations provide both operational support and community engagement expertise. Key contributions include:

- Safe Blood for Africa Foundation and AfSBTsupport training programs, facility audits, and the implementation of quality management systems.
- Médecins Sans Frontières operates emergency transfusion services in conflict zones or outbreak-affected regions, offering not only clinical support but also technical input for system recovery.
- NGOs such as Blood: Water and Partners for Health collaborate with local health workers to build awareness, mobilize donors, and integrate blood donation into existing public health initiatives (e.g., HIV care or maternal health)[36].

### Multi-stakeholder success stories

Several African countries illustrate the impact of effective partnerships:

- In Rwanda, a partnership between the Ministry of Health, WHO, and Global Fund led to the creation of a national transfusion service, which achieved 100% screening coverage for TTIs by 2021.
- In Nigeria, collaboration between the NBTS PEPFAR, and civil society organizations resulted in a 60% increase in voluntary blood donation in pilot states between 2018 and 2022.
- Botswana’s blood system transformation was bolstered by WHO guidance and PEPFAR funding, enabling the introduction of digital traceability platforms and training of over 1,000 staff in transfusion safety practices[37].

## Sustainable reforms in African blood transfusion systems: the power of policy and political will

Sustainable transformation in blood transfusion services across Africa requires more than temporary fixes – it demands structural reform, policy innovation, and, above all, political commitment. In several countries, the evolution of blood systems from fragmented and donor-dependent models into coordinated, high-functioning services has been driven by long-term policy changes, underpinned by national leadership and strategic partnerships. Two noteworthy examples – Rwanda’s centralized national system and Ghana’s model of public-private collaboration – offer compelling insights into how politically-backed reforms can shape more resilient and equitable blood services.

## Rwanda: centralization as a pathway to quality and equity

Rwanda stands out in the region for its bold and decisive move toward centralizing its blood transfusion services under the leadership of the Ministry of Health. In the aftermath of the 1994 genocide, the country faced severe health infrastructure deficits, including a highly fragmented blood system. Recognizing the critical importance of safe blood – especially for maternal health, trauma care, and infectious disease management – Rwanda undertook sweeping reforms to standardize, professionalize, and integrate its transfusion network. By establishing the Rwanda NCBT and organizing regional blood banks into a single, coordinated entity, the country ensured consistent protocols for donor recruitment, screening, storage, and distribution. This centralization enabled efficient allocation of blood to hospitals based on real-time needs, reduced wastage, and improved traceability. Importantly, these reforms were not merely technical. They were anchored in a national policy framework, supported by sustained investment and political will. The Rwandan government committed resources to infrastructure, staff training, and community sensitization campaigns, leading to a steady rise in VNRBDs. As a result, Rwanda now boasts one of the safest and most accessible blood supplies in the region, with near-universal screening for TTIs and a reliable emergency supply system that serves both urban and rural populations^[35,36]^.

## Ghana: leveraging public-private partnerships for sustainability

Ghana has taken a different, but equally impactful, approach to reform – —leveraging public-private partnerships to bridge funding gaps, enhance logistics, and extend donor outreach. While Ghana’s National Blood Service operates under the Ministry of Health, its most notable advances have come through collaborative models involving private logistics firms, civil society, and international donors. One such initiative is the Zipline drone delivery system, developed through a partnership between the government and a private tech company. This innovation has revolutionized the way blood and medical supplies are delivered to remote health facilities, particularly in the country’s northern and forested regions. With rapid response times and GPS tracking, the drone service ensures that blood units can be delivered within 30 minutes to facilities that previously waited hours – or even days – due to poor road access. Furthermore, Ghana’s engagement with private media and educational institutions has amplified public awareness campaigns, while partnerships with NGOs have expanded mobile collection drives and supported cold chain development. What makes Ghana’s reform strategy noteworthy is its multi-stakeholder nature and the government’s proactive role in policy coordination, regulation, and accountability. Political will has been essential in facilitating legal frameworks for partnerships, ensuring data transparency, and aligning donor efforts with national goals^[36,37]^.

## Lessons in leadership and longevity

Both Rwanda and Ghana exemplify how political leadership and targeted policy changes can drive long-lasting improvements in blood transfusion systems. Their experiences show that sustainable reform does not require a one-size-fits-all model, but it does demand:

- A clear national vision for blood safety and access.
- Integration of blood services into health system planning.
- Long-term investment in infrastructure and human resources.
- Willingness to innovate through partnerships and technology.

These cases provide a valuable blueprint for other African nations seeking to strengthen their transfusion services. When reforms are locally owned, strategically implemented, and politically supported, they not only improve blood safety and availability but also contribute to broader goals of health equity, system resilience, and public trust[37].

## A multi-level intervention model to strengthen blood transfusion services

Addressing the complex challenges of blood transfusion systems in Africa demands a comprehensive, multi-level approach that integrates capacity building, technological innovation, and community outreach. Effective blood services must not only ensure safety and quality but also bridge geographic and systemic gaps that limit access – particularly in rural and underserved regions. Two countries, Namibia and Uganda, illustrate how combining health worker training, digital data management, and decentralized mobile collection units can produce measurable improvements in blood availability and safety.

## Capacity building through health worker training

At the foundation of any robust blood transfusion system lies a skilled and knowledgeable workforce. Namibia has invested heavily in targeted training programs for health professionals involved in blood collection, testing, storage, and transfusion. These initiatives emphasize adherence to standardized protocols, quality assurance, and patient safety. Through partnerships with international bodies such as WHO and local universities, Namibia has developed continuous education modules and certification systems, enabling staff to stay current with evolving technologies and guidelines. This ongoing professional development not only improves procedural competence but also builds confidence and accountability within blood services[35].

## Harnessing electronic data systems for supply chain management

A critical advancement in both Namibia and Uganda has been the adoption of electronic data systems that track blood donation, inventory, and distribution in real time. These digital platforms provide centralized visibility into supply and demand patterns, enabling more efficient resource allocation and minimizing wastage. In Namibia, the introduction of a national blood information management system has streamlined donor database management, transfusion requests, and laboratory results. This system also facilitates rapid identification of rare blood types and supports hemovigilance efforts by flagging adverse transfusion events. Uganda has similarly implemented an electronic blood tracking system connected to both urban blood banks and peripheral health facilities. By integrating mobile technology, Uganda’s system allows for timely updates and alerts, ensuring that blood stocks are replenished before critical shortages occur[36].

## Decentralized mobile collection units to reach remote populations

One of the most significant barriers to adequate blood supply in Africa is the challenge of accessing donors in remote or rural areas. To overcome this, both Namibia and Uganda have deployed decentralized mobile blood collection units that travel to underserved communities. These mobile units, often equipped with refrigeration and testing facilities, enable blood collection drives outside traditional health facilities, reducing geographic barriers for voluntary donors. In Namibia, mobile clinics have been vital in reaching nomadic populations and rural settlements, resulting in increased donor diversity and volume. Uganda’s mobile units are integrated into broader community health outreach programs, combining blood donation with health education and screening for common illnesses. This holistic approach fosters community trust and engagement, which is essential for sustainable donor recruitment[37].

## Synergistic impact of multi-level interventions

The combination of trained personnel, real-time data systems, and mobile outreach creates a synergistic effect – strengthening each link in the blood supply chain from donor recruitment to transfusion safety. These interventions help countries like Namibia and Uganda build resilient and responsive blood services, capable of adapting to fluctuating demands and resource constraints. By scaling such multi-level models and tailoring them to local contexts, other African countries can similarly enhance blood availability, reduce transfusion-related risks, and improve patient outcomes – ultimately contributing to stronger health systems and equitable care access[37].

## Integrating blood safety into maternal health and infectious disease programs: aligning with national priorities and the SDGs

In many African countries, blood transfusion services are most urgently needed in the context of maternal health emergencies and the management of infectious diseases such as HIV, malaria, and tuberculosis. Recognizing this, recent strategic efforts emphasize the integration of blood safety and availability within broader national health priorities, thereby maximizing impact and resource efficiency.

## Linking blood safety to maternal health

Maternal mortality remains a significant challenge across Africa, with postpartum hemorrhage (PPH) as one of the leading causes of maternal death. Effective management of PPH frequently requires timely access to safe blood transfusions. Consequently, blood services are increasingly being embedded within national maternal and neonatal health programs to ensure that blood availability is prioritized alongside antenatal care, skilled birth attendance, and emergency obstetric services. For example, several countries have developed protocols that guarantee immediate blood supply for obstetric emergencies, supported by strengthened referral networks and emergency transport systems. Training programs for midwives and obstetricians now routinely include components on transfusion indications, cross-matching, and adverse reaction monitoring, fostering closer collaboration between blood banks and maternity wards[35].

## Integration with infectious disease control

Beyond maternal health, blood safety is critical in managing infectious diseases endemic to the region. The prevalence of TTIs – notably HIV, hepatitis B and C, and syphilis – makes rigorous donor screening and post-donation testing indispensable. National HIV control programs often incorporate blood safety measures as part of comprehensive prevention strategies. This includes aligning donor recruitment campaigns with HIV awareness and testing services, thereby reducing risks and promoting VNRBD from low-risk populations. Countries like Kenya and Tanzania have successfully integrated blood transfusion services into their infectious disease frameworks, ensuring coordinated surveillance, data sharing, and community education efforts that benefit both sectors[36].

## Aligning with sustainable development goals

These integration efforts directly support progress toward multiple **Sustainable Development Goals**, particularly:

- **SDG 3 (Good Health and Well-being):** By reducing maternal mortality, improving outcomes for trauma and surgical patients, and preventing transfusion-transmitted infections, safer blood transfusion services contribute to healthier populations.
- **SDG 10 (Reduced Inequalities):** Ensuring equitable access to safe blood across urban and rural areas addresses disparities in health care availability.
- **SDG 17 (Partnerships for the Goals):** Integration fosters multisectoral collaboration between ministries of health, donor agencies, NGOs, and community groups.

## Policy implications and future directions

Embedding blood safety within maternal and infectious disease programs encourages governments to prioritize transfusion services in national health agendas, unlocking resources and political support. It also facilitates monitoring and evaluation through existing health information systems, improving accountability and service quality. Ultimately, such alignment ensures that blood transfusion is not a standalone service but a vital, integrated component of comprehensive health care delivery – amplifying its impact on population health and accelerating progress toward global health targets[37].

## Culturally sensitive donor recruitment and addressing economic inequities

Effective blood donor recruitment in Africa requires more than just outreach – it demands a deep understanding of local cultural contexts, beliefs, and socioeconomic realities that influence people’s willingness and ability to donate. To ensure a safe and reliable blood supply, transfusion services must adopt culturally sensitive strategies that build trust, respect community norms, and actively reduce economic barriers that disproportionately affect rural and underserved populations.

## Respecting cultural beliefs and building trust

In many African communities, cultural perceptions and traditional beliefs around blood and health strongly shape attitudes toward donation. Myths about blood loss causing weakness, spiritual harm, or community stigma can deter potential donors. Successful recruitment efforts therefore prioritize engagement with community leaders, traditional healers, and local influencers who can endorse blood donation within culturally acceptable frameworks. For example, in some regions, blood drives are integrated with local festivals, religious gatherings, or community meetings, making donation a collective, normalized activity rather than an isolated medical procedure. Educational campaigns employ locally relevant languages, storytelling, and culturally resonant messaging that emphasize solidarity, altruism, and the life-saving impact of donating blood[38].

## Addressing economic barriers: subsidized transport and accessibility

Economic inequities also play a major role in limiting donor participation, especially for individuals in remote or rural areas who face transportation challenges and direct costs associated with reaching donation centers. Recognizing this, several programs have introduced subsidized or free transport services to facilitate access to fixed or mobile blood collection sites. By alleviating the financial burden of travel, these initiatives not only increase donor turnout but also promote equitable access, ensuring that underserved populations are not excluded due to economic constraints. In countries like Uganda and Malawi, partnerships with local governments and NGOs have enabled provision of transport vouchers or organized community shuttle services during blood donation campaigns[27].

## Community sensitization campaigns: informing and empowering

Complementing these logistical supports are community sensitization campaigns that focus on raising awareness about the importance of blood donation and dispelling misconceptions. Through radio programs, local theater, school outreach, and social media, these campaigns reach diverse audiences and encourage voluntary, non-remunerated donation as a shared civic responsibility. Community health workers play a vital role in these efforts, serving as trusted messengers who can address individual concerns, identify eligible donors, and provide follow-up support. Targeted engagement with youth groups, women’s associations, and workplaces further expands the donor base by fostering a culture of donation that is inclusive and sustained over time[38].

## Toward inclusive and equitable blood donation systems

Together, culturally sensitive recruitment strategies and economic support mechanisms create an enabling environment where more individuals can participate safely and willingly in blood donation. These approaches are essential for developing equitable blood transfusion systems that reflect the diversity and needs of African communities, ensuring no one is left behind due to cultural misunderstandings or financial hardship. By valuing local knowledge, respecting cultural nuances, and addressing socioeconomic barriers, transfusion services can build stronger, more resilient donor pools that support improved health outcomes across the continent^[27,39]^.

## Conclusion

Blood transfusion is an essential component of health care in Africa, providing life-saving support for patients suffering from severe anemia, maternal hemorrhage, trauma, and blood disorders. While significant advancements have been made in donor recruitment, blood screening technologies, infrastructure development, and policy reforms, numerous barriers continue to hinder the effectiveness of blood transfusion services. Low voluntary blood donation rates, high prevalence of TTIs, inadequate infrastructure, financial constraints, and shortages of skilled personnel remain critical challenges that must be addressed to improve blood safety and availability. To ensure a sustainable and efficient blood transfusion system, African countries must adopt a multi-faceted approach that includes increasing public awareness, strengthening regulatory frameworks, and investing in modern technologies. Expanding voluntary blood donation programs, enhancing screening and testing capabilities, and leveraging innovations such as drone delivery and mobile applications can significantly improve blood supply management. Additionally, fostering regional collaborations and securing long-term funding will be crucial in overcoming logistical and financial barriers.

## Data Availability

Not applicable as this a Narrative Review.

## Research registration unique identifying number (UIN)

Not applicable as this a Narrative Review.
